# Down‐regulation of *OsSPX1* caused semi‐male sterility, resulting in reduction of grain yield in rice

**DOI:** 10.1111/pbi.12527

**Published:** 2016-01-25

**Authors:** Kang Zhang, Qian Song, Qiang Wei, Chunchao Wang, Liwei Zhang, Wenying Xu, Zhen Su

**Affiliations:** ^1^ State Key Laboratory of Plant Physiology and Biochemistry College of Biological Sciences China Agricultural University Beijing China

**Keywords:** *OsSPX1*, rice, pollen fertility, grain yield, expression profiling

## Abstract

*OsSPX1*, a rice SPX domain gene, involved in the phosphate (Pi)‐sensing mechanism plays an essential role in the Pi‐signalling network through interaction with *OsPHR2*. In this study, we focused on the potential function of *OsSPX1* during rice reproductive phase. Based on investigation of *OsSPX1* antisense and sense transgenic rice lines in the paddy fields, we discovered that the down‐regulation of *OsSPX1* caused reduction of seed‐setting rate and filled grain number. Through examination of anthers and pollens of the transgenic and wild‐type plants by microscopy, we found that the antisense of *OsSPX1* gene led to semi‐male sterility, with lacking of mature pollen grains and phenotypes with a disordered surface of anthers and pollens. We further conducted rice whole‐genome GeneChip analysis to elucidate the possible molecular mechanism underlying why the down‐regulation of *OsSPX1* caused deficiencies in anthers and pollens and lower seed‐setting rate in rice. The down‐regulation of *OsSPX1* significantly affected expression of genes involved in carbohydrate metabolism and sugar transport, anther development, cell cycle, etc. These genes may be related to pollen fertility and male gametophyte development. Our study demonstrated that down‐regulation of *OsSPX1* disrupted rice normal anther and pollen development by affecting carbohydrate metabolism and sugar transport, leading to semi‐male sterility, and ultimately resulted in low seed‐setting rate and grain yield.

## Introduction

Rice (*Oryza sativa*), one of the major food staples for the world's population, is a model monocot plant for molecular biological study and a model crop for agronomical improvement. The rice grain yield is affected by many genetic and environmental factors, such as photosynthesis ability, nutrient efficiency, processes of pollination and fertilization, biotic and abiotic stresses, etc. Pollen fertility is a critical factor for rice yield. Dozens of genes are involved in anther and pollen development in rice and *Arabidopsis*, including anther cell differentiation, meiosis and pollen development (Wilson and Zhang, 2009). The development of pollen and anther requires nutrients such as sugars and lipids from source organs to support pollen development and maturation (Goetz *et al*., 2001). Carbon Starved Anther (*CSA*), encoding a MYB transcription factor, is involved in sugar partitioning and the *csa* mutant showed low carbohydrate level in later anthers with male sterility (Zhang *et al*., 2010). Defective Pollen Wall (DPW) is a fatty acyl reductase, and the mutant *dpw* showed defective anther development and degenerated pollen grains with an irregular exine (Shi *et al*., 2011). The tapetum degeneration triggered by a programmed cell death (PCD) process provides cellular contents supporting pollen wall formation (Wu and Cheun, 2000). The *TDR* (Tapetum Degeneration Retardation) encodes a putative transcription factor with a bHLH domain. In the *tdr* mutant, the tapetum PCD was retarded in the anther with aborted pollen development (Li *et al*., 2006). The *Arabidopsis* ortholog *AMS* (Aborted Microspores) showed similar function (Xu *et al*., 2010). UDP‐glucose pyrophosphorylase (UGPase) is a key enzyme in carbohydrate metabolism, producing UDP‐glucose for sucrose synthesis in leaves. In *Arabidopsis pho1* mutants, *Ugp* was found to be up‐regulated under conditions of phosphate deficiency (Ciereszko *et al*., 2001). Rice *Ugp1* is essential for pollen callose deposition, and the *Ugp1*‐silenced plants showed thermosensitive male sterility (Chen *et al*., 2007). Rice *OsUgp2* is a pollen‐preferential gene and plays a critical role in starch accumulation during pollen maturation (Mu *et al*., 2009).

Phosphorus is one of the major mineral nutrients for plant growth and development. Phosphate (Pi) has regulatory function in reactions of photosynthetic carbon metabolism and is involved in the photosynthetic carbon assimilation and carbon partitioning processes between starch and sucrose (Rao, 1996), through the operation of the Pi translocator to facilitate a rapid exchange of Pi, triose‐P and 3‐phosphoglyceric acid (PGA) (Flugge, 1995). The Pi concentration inside and outside the chloroplast could affect the photosynthetic carbon reduction and control the balance between starch in chloroplast and sucrose in the cytosol (Rao, 1996). The dynamic interactions between sink and source tissues affected the response of photosynthesis to phosphate limitation (Pieters *et al*., 2001).

There exists close relationship between phosphate signalling and rice reproductive development. Rice plants can accumulate abundant Pi in vegetative organs such as leaves at the early developmental stage and transport the Pi stored in the leaves to reproductive organs such as panicle at the late developmental stage (Marschner, 1995). The rice phosphate transporter gene *OsPT8* is involved in Pi translocation from vegetative organs to reproductive organs in rice. The suppression of *OsPT8* resulted in lower seed‐setting rate, higher phosphorus content in the panicle axis and decreased phosphorus content in unfilled grain hulls (Jia *et al*., 2011). Under high Pi level, overexpressing *OsPHR2* up‐regulated some Pi transporter genes and suppressed the growth parameters during harvest stage, for example lower seed‐setting rate, lower tiller number and grain number. In particular, the *OsPHR2‐Ov1* transgenic line showed disordered male reproductive organs such as twisted anther structures, few pollen grains and low pollen viability (Zhou *et al*., 2008). The *ltn1* (*OsPHO2*) mutant significantly reduced tiller number and fertility compare to WT (Hu *et al*., 2011). *OsSPX1*, as one of the Pi‐dependent inhibitors of *OsPHR2* activity in rice (Wang *et al*., 2014), is involved in the Pi starvation signalling network related to *OsPHR2* and *OsPHO2* (Liu *et al*., 2010), but there is no any report about the effect of *OsSPX1* on pollen development and grain yield.

In plants, many SPX domain proteins (with SPX domain, named after the SYG1/Pho81/XPR1 proteins) were identified to be involved in the phosphate‐related signal transduction pathway and regulation pathways. For example, the AtPHO1 (At3g23430) protein is involved in ion transport in *Arabidopsis* (Hamburger *et al*., 2002; Stefanovic *et al*., 2007; Wang *et al*., 2004, 2008); the AtSPX family proteins were considered as part of the phosphate‐signalling pathways controlled by PHR1 and SIZ1 (Duan *et al*., 2008); AtSPX1 was identified as the inhibitor of PHR1 and the SPX1/PHR1 interaction was Pi‐dependent (Puga *et al*., 2014); the *OsSPX1* is involved in the Pi‐sensing mechanism (Liu *et al*., 2010); OsSPX1 and OsSPX2 are Pi‐dependent inhibitors of OsPHR2 through the protein‐protein interaction and are involved in the Pi‐sensing process of rice (Wang *et al*., 2014); *Os*SPX*3* and *OsSPX5* are redundant genes negatively regulating root‐to‐shoot Pi translocation and restored phosphate balance under phosphate starvation (Shi *et al*., 2014); OsSPX4 protein is responsive to Pi concentration and regulates the activity of OsPHR2 with the protein–protein interaction (Lv *et al*., 2014). Besides the essential role of SPX domain proteins involved in phosphate signalling, some of them have other key functions. For example, a PHO1 family protein, SHB1, contains an N‐terminal SPX domain and a C‐terminal EXS domain and was reported to specifically regulate blue‐light responses and/or possibly red and far‐red light responses in *Arabidopsis* (Kang and Ni, 2006). Both SPX and EXS domains likely anchor SHB1 to a protein complex, and the SPX domain is critical for SHB1 signalling, which plays dual roles in photoperiodic and autonomous flowering (Zhou and Ni, 2009; 2010). Furthermore, SHB1 was identified as a positive regulator of *Arabidopsis* seed development that affected both cell size and number (Zhou *et al*., 2009).

Our previous study reported that constitutive overexpression of *OsSPX1* in tobacco and *Arabidopsis* plants caused the improvement of cold tolerance with decreasing total leaf Pi (Zhao *et al*., 2009) and down‐regulation of *OsSPX1* caused transgenetic rice high sensitivity to cold and oxidative stresses in seedling stage (Wang *et al*., 2013). In the generated *OsSPX1* transgenic rice lines, we observed the *Ubi::OsSPX1*‐antisense (down‐regulation of *OsSPX1*) lines showed significantly lower seed‐setting rate in the reproductive stage, which may be correlated with semi‐male sterility in the *Ubi::OsSPX1*‐antisense lines. In this study, we focused on the effect of *OsSPX1* on pollen development and grain yield with the *OsSPX1* antisense and sense transgenic rice lines. We conducted rice whole‐genome GeneChip to elucidate the possible molecular mechanism and identified the downstream key genes involved in the relationship between *OsSPX1* and rice pollen development and grain yield. This work on *OsSPX1* may aid understanding of the possible novel functions of *OsSPX1* and be greatly beneficial for improving plant growth and crop grain yield.

## Results

### Antisense of *OsSPX1* caused reduction of rice grain yield in paddy fields

To investigate the traits during reproductive stages, the *Ubi::OsSPX1‐antisense* transgenic, *Ubi::OsSPX1‐sense* transgenic and wild‐type (WT) plants were planted in paddy fields. The construction of *OsSPX1* transgenic lines was described in our previous work (Wang *et al*., 2013). The expression levels of *OsSPX1* in the mature leaves of WT and transgenic rice lines were shown in Figure S1. *OsSPX1* were significantly suppressed in *Ubi::OsSPX1‐antisense* transgenic lines (lines A1 and A2) compared to WT plants (left chart in Figure S1), and constitutively and more strongly expressed in *Ubi::OsSPX1‐sense* transgenic lines (S1 and S2) than WT plants (right chart in Figure S1).

**Figure 1:**
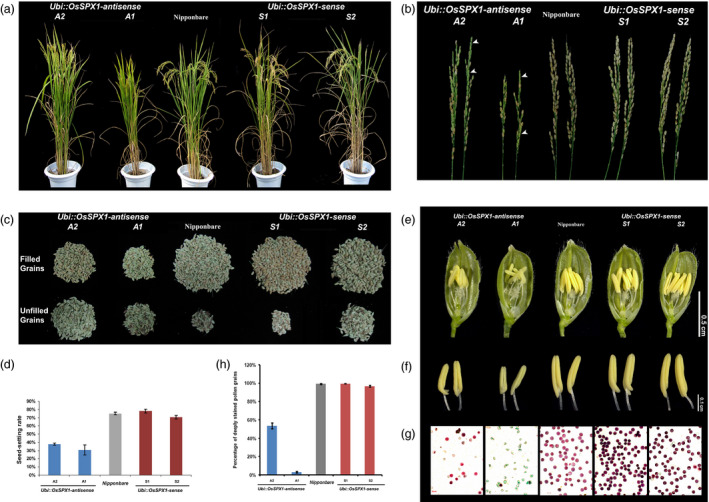
Agricultural traits and anther phenotypes of WT, *Ubi::OsSPX1‐antisense* and *Ubi::OsSPX1‐sense* transgenic rice lines. (a) Comparison of *Ubi::OsSPX1‐antisense* transgenic lines (lines A1, and A2, on the left), *Ubi::OsSPX1‐sense* transgenic lines (lines S1, and S2, on the right) with the wild‐type (WT) rice at the grain‐filling stage grown in paddy fields. (b) The harvested panicles of *Ubi::OsSPX1‐antisense* transgenic lines (lines A1, and A2, on the left), *Ubi::OsSPX1‐sense* transgenic lines (lines S1, and S2, on the right) and the wild‐type (WT) rice. (white arrows indicate empty seeds). (c) The total grains of the plant of *Ubi::OsSPX1‐antisense* transgenic lines (lines A1, and A2, on the left), *Ubi::OsSPX1‐sense* transgenic lines (lines S1, and S2, on the right) and the wild‐type (WT) rice. (d) Comparison of seed‐setting rate of *Ubi::OsSPX1‐antisense* transgenic lines (in blue bars), *Ubi::OsSPX1‐sense* transgenic lines (in red bars) with the WT (in grey bars) rice. (e) Comparison of spikelets after removing half of the lemma and palea, Bar = 0.5 cm. (f) Comparison of anthers from *Ubi::OsSPX1‐antisense* transgenic lines (lines A1, and A2, on the left), *Ubi::OsSPX1‐sense* transgenic lines (lines S1, and S2, on the right) and the wild‐type (WT) rice, Bar = 0.1 cm. (g) Comparison of pollen grains stained with Alexander's solution, Bar = 50 μm. (h) Percentage of stained pollen grains.

During the ripening phases, we compared the traits related to grain yield among the *Ubi::OsSPX1‐antisense* transgenic lines, *Ubi::OsSPX1‐sense* transgenic lines and WT plants. The *Ubi::OsSPX1‐antisense* transgenic lines (A1 and A2) exhibited lower seed‐setting rate and filled grain number (Figure 1). The panicles in lines A1 and A2 were straight while those in *Ubi::OsSPX1‐sense* transgenic lines (S1 and S2) and WT plants were bent (Figure 1a). The harvested panicles of A1 and A2 lines were not mature, and the panicles of A1 were much smaller than those of WT, S1 and S2 (Figure 1b, the empty seeds in lines A1 and A2 were highlighted by white arrows). We separated the filled and unfilled grains of individual plants of each lines, and noticed the seed‐setting ratio was significantly lower in A1 and A2 than those in WT, S1 and S2 (Figure 1c). The seed‐setting rate in A1 and A2 lines was at least 50% lower compared to WT, S1 and S2 lines (the *t*‐test results were significant, *P*‐values were lower than 0.01). The lower seed‐setting rate led to a reduction of grain yield in the *Ubi::OsSPX1‐antisense* transgenic rice plants (Figure 1d), and the phenotype was stable for later generations (Figure S2).

### Antisense of *OsSPX1* caused lower pollen viability with disordered pollen and anther

The pollen fertility is a critical factor for rice grain yield. We proposed that down‐regulation of *OsSPX1* may affect the anther and pollen development in rice. We discovered that the *Ubi::OsSPX1‐antisense* transgenic lines showed semi‐male sterility during anther and pollen development. Figure 1e,f showed the anther phenotype of *Ubi::OsSPX1‐antisense* transgenic lines, *Ubi::OsSPX1‐sense* transgenic lines and WT plants in paddy fields. During the heading stage, the spikelets from *Ubi::OsSPX1‐antisense* transgenic lines had smaller pale‐yellow anthers compared to the normal yellow anthers from *Ubi::OsSPX1‐sense* transgenic lines and WT (Figure 1e,f). Unlike mature pollen of WT and *Ubi::OsSPX1‐sense* transgenic lines, large proportion of pollen of *Ubi::OsSPX1‐antisense* transgenic lines could not be stained by Alexander's solution (Figure 1g,h). These results indicated that the *Ubi::OsSPX1‐antisense* transgenic lines lacked normal mature pollen grains and this might cause the lower seed‐setting rate and filled grain number. The pollen viability of different transgenic lines and WT rice plants in the heading stage was correlated with the seed‐setting rate at the harvest stage (Figure 1d,h).

**Figure 2:**
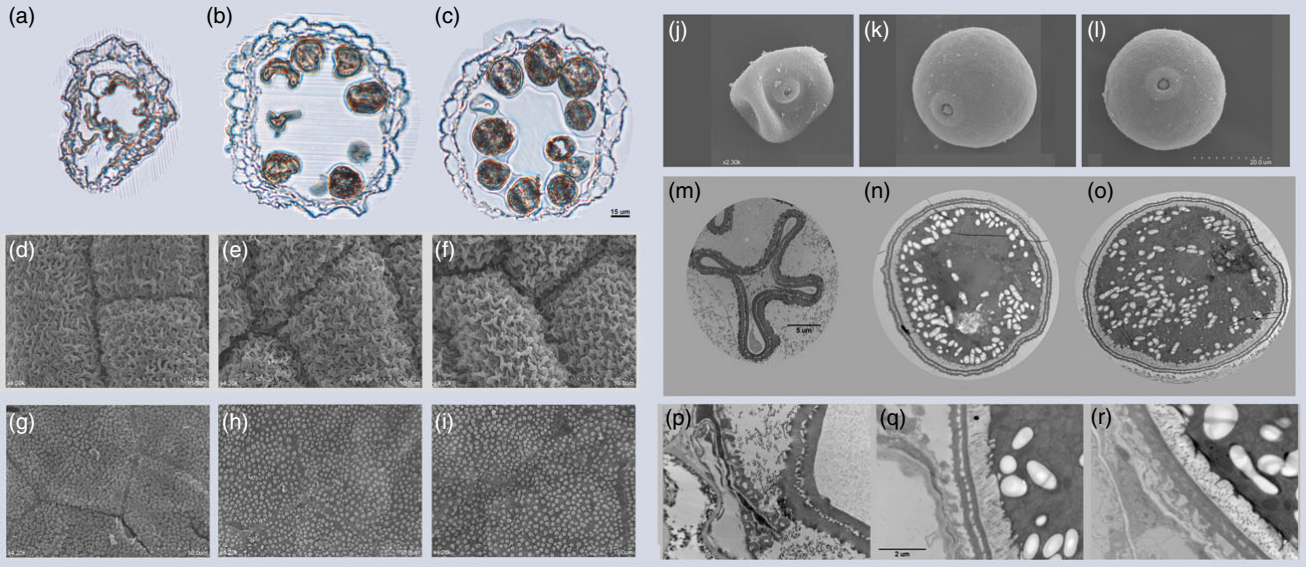
Pollen phenotypes among WT, *Ubi::OsSPX1‐antisense* and *Ubi::OsSPX1‐sense* transgenic rice lines. (a–c) Cross section of a single locule in stage 12 for A1, WT and S1, Bar = 15 μm. (d–f) Scanning electron microscopy analysis of the anther surface of A1, WT and S1 at stage 12, Bar = 10 μm. (g–i) Scanning electron microscopy analysis of the inner surface of the anther wall layers at stage 12 for A1, WT and S1, Bar = 10 μm. (j–l) Scanning electron microscopy analysis of the pollen grain at stage 12 for A1, WT and S1, Bar = 20 μm. (m–o) Transmission electron micrograph of the pollen grain at stage 12 for A1, WT and S1, Bar = 5 μm. (p–r) Transmission electron micrograph of the pollen wall at stage 12 for A1, WT and S1, Bar = 2 μm.

Furthermore, we examined the anther development by light microscopy and analysed the anther and pollen by scanning electron microscopy (SEM) and transmission electron microscopy (TEM) (Figure 2). At stage 12 of anther development (Zhang *et al*., 2011), for WT and *Ubi::OsSPX1‐sense* transgenic line S1, the pollen grains were full of starch and lipids and the tapetums were almost degenerated; whereas in the anther of *Ubi::OsSPX1‐antisense* transgenic line A1, the microspores were hardly visible and the anther locule was almost empty (Figure 2a–c).

The cuticle on the exterior of the anthers of *Ubi::OsSPX1‐antisense* transgenic line was not as well formed as those on the anther outer surface of the WT and *Ubi::OsSPX1‐sense* transgenic line (Figure 2d–f). The Ubisch bodies on the inner locule surface were also different (Figure 2g–i), granular for WT and *Ubi::OsSPX1‐sense* transgenic line S1 and shrunken for *Ubi::OsSPX1‐antisense* transgenic line A1. In addition, the pollen grains of WT and S1 had a smooth and particulate exine pattern, whereas the pollen grains of A1 appeared severely shrunken and empty (Figure 2j–l).

Further TEM observations showed consistent phenomena. At stage 12, the pollen grains of WT and *Ubi::OsSPX1‐sense* transgenic line S1 were full of storage materials, with more starch granules and lipids in S1 and WT, whereas the *Ubi::OsSPX1‐antisense* transgenic line A1 pollen grains were collapsed with almost no accumulated storage materials (Figure 2m–o), the storage materials in the pollen of A1, WT and S1 were correlated with the Alexander staining results for their pollen (Figure 1g). Compared with those of the WT and S1, the pollen wall of A1 showed thicker exine layer (both tectum and nexines) and almost no intine layer (Figure 2p–r).

### Transcriptome map of anthers from *OsSPX1* transgenic lines and WT plants

**Figure 3:**
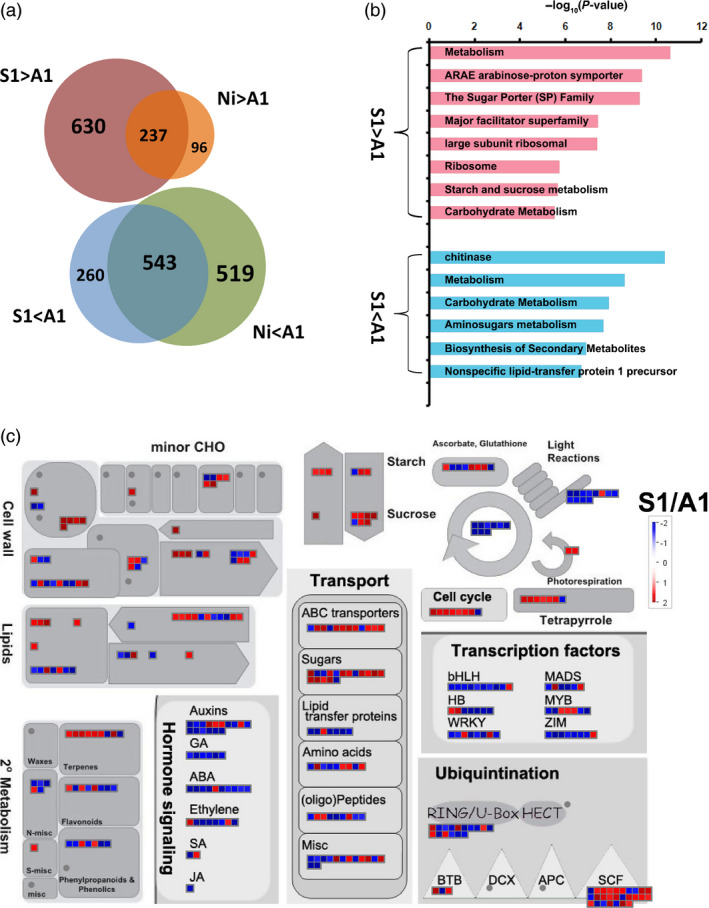
Venn diagrams analysis and MapMan view of differential expression probe sets in the anthers between line A1 and S1, and between line A1 and WT(Ni). (a) Venn diagrams illustrate the differential expression probe sets in the anthers among transgenic line A1, S1 and Ni (WT). (b) GeneBins analysis of the differential expression probe sets in the anthers between line A1 and S1. (c) MapMan view of the differential expression probe sets in the anthers between line A1 and S1. Fold changes are shown in colour, red boxes indicate up‐regulated in S1, and blue boxes indicate up‐regulated in line A1.

We conducted rice whole‐genome GeneChip to elucidate the possible molecular mechanism underlying the down‐regulation of *OsSPX1* in causing deficiencies in anther and pollen and lower seed‐setting rate in rice. There were nine anther samples in total: three independent biological samples each were collected from *Ubi::OsSPX1‐antisense* transgenic line A1, *Ubi::OsSPX1‐sense* transgenic line S1 and WT (Nipponbare) plants during heading stage. We mainly focused on the differentially expressed probe sets between line A1 (*Ubi::OsSPX1‐antisense*) and line S1 (*Ubi::OsSPX1‐sense*), and between line A1 and WT. With ANOVA test (*P *≤ 0.05) and 1.5‐fold change as cut‐off, there were 867 probe sets significantly higher expressed in anthers of line S1 than A1, and 803 probe sets were significantly lower expressed in line S1 than A1; 333 probe sets were significantly higher expressed in WT than in line A1 anthers, and 1062 probe sets significantly lower expressed in WT than A1 (shown in Figure 3a, the detailed information for each probe set was listed in Table S1). The Venn diagram in Figure 3a illustrated the intersection of probe sets between each groups, there were 237 overlapped probe sets more highly expressed both in line S1 and WT plants compared to line A1 anthers, and 543 overlapped probe sets were less expressed both in line S1 and WT plants compared to A1.

We classified these differentially expressed genes using GeneBins (Goffard and Weiller, 2007). Figure 3b highlighted several classes (BINs) of genes significantly more highly expressed in line S1 than in A1, including sugar porter (SP) family, starch and sucrose metabolism, etc. As to the genes significantly more highly expressed in line A1, the enriched BINs included chitinase, lipid‐transfer protein, etc. Further analysis with MapMan tool (Thimm *et al*., 2004) showed that there were several biological metabolism processes and large gene families in these differentially expressed genes (shown in Figure 3c). The genes related to cell wall, starch–sucrose, cell cycle and sugar transporter were highly expressed in line S1, whereas some transcription factor genes (such as WRKY, BHLH, ZIM, etc.) and hormone response genes (related to GA, ABA, ethylene, etc.) were highly expressed in line A1.

**Figure 4:**
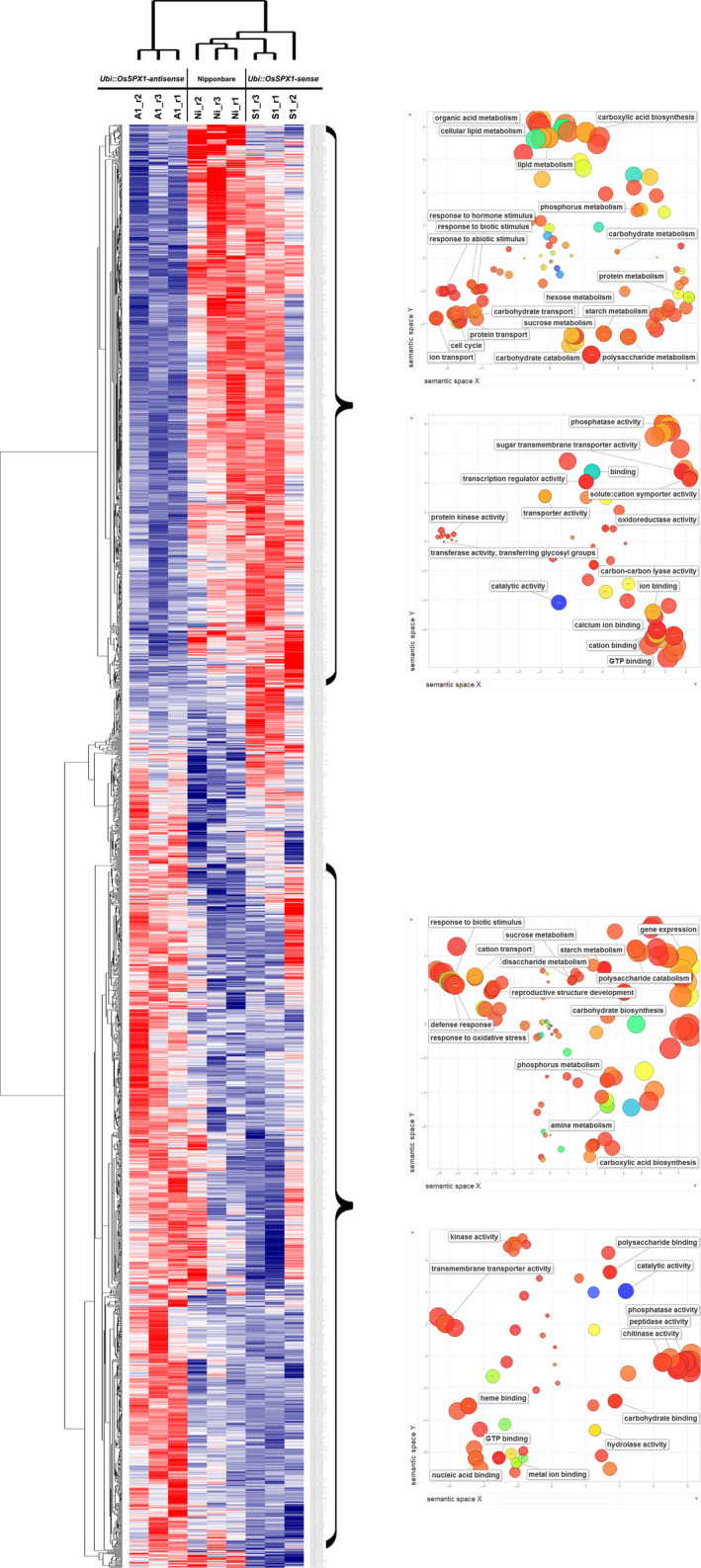
Cluster and gene ontology (GO) analysis of the differentially expressed probe sets in the anthers among transgenic line A1, S1 and WT(Ni). The overview hierarchical cluster result of 2403 probe sets showing differential expression in anthers among *Ubi::OsSPX1‐sense* transgenic line (S1), WT(Ni) and *Ubi::OsSPX1‐antisense* transgenic lines (A1); the red (high) and blue (low) colours represent the relative expression level across the samples. The marked groups represent these probe sets specifically down‐regulated in line A1 (upper group) or up‐regulated in line A1 (lower group). The charts on the right represent the enriched GO terms in the probe sets belonging to the marked group.

To further identify the co‐expressed probe sets with similar expression patterns in the transgenic and WT plants, both self‐organized mapping (SOM) and hierarchical methods were used for clustering the 2403 differentially expressed probe sets among line S1, line A1 and WT (listed in Table S1). A colour heat map represented the relative expression level of each probe set across the nine anther samples (shown in Figure 4, from left to right, three replicates each of line A1, WT and line S1). These probe sets could be grouped into multiple clusters, and we focused on two of them: the top one representing the probe sets lower expressed in line A1 anther samples, and the bottom one representing the probe sets higher expressed in line A1. We applied Gene Ontology (GO) analysis‐using agriGO (Du *et al*., 2010) and REVIGO (Supek *et al*., 2011)‐to the selected clusters (shown in the right side of Figure 4). For the top cluster representing the probe sets lower expressed in line A1 anther samples, the enriched GO terms were mainly related to sucrose and starch metabolism (FDR *P*‐value: 3.40E‐16 and 7.20E‐10, respectively), carbohydrate transport (FDR *P*‐value: 4.10E‐05), sugar:hydrogen symporter (FDR *P*‐value: 6.10E‐04), etc. As to the probe sets highly expressed in line A1 anther samples, the enriched GO terms included defence response (FDR *P*‐value: 9.50E‐05), chitinase activity (FDR *P*‐value: 2.40E‐07), etc.

Finally, we highlighted several enriched GO terms and functional groups for either lower or higher expressed in *Ubi::OsSPX1‐antisense* transgenic lines (Figures 3 and 4) based on the results including gene cluster analysis, GeneBins, MapMan, and GO enrichment analysis. Some important genes were also highlighted in Table 1 and Table S3, were involved in sucrose and starch metabolism, sugar transporter, anther development, cell cycle and microtubule‐based process, chitinase, and phenylalanine metabolism.

**Table 1:** Selected differentially expressed probe sets related to enriched function terms

Probe set ID	S1 vs. A1		Ni vs. A1		Locus ID	Annotation
	*P*‐value	Fold change	*P*‐value	Fold change		
Starch and sucrose metabolism						
Os.11216.1.S1_at	1.70E‐02	1.62	3.07E‐02	1.54	LOC_Os03g55090	Alpha‐1,4 glucan phosphorylase, L isozyme
Os.57438.1.S1_at	3.57E‐02	1.76	3.70E‐02	1.76	LOC_Os02g52700	Alpha‐amylase precursor
Os.52873.1.S1_a_at	3.30E‐02	1.54	4.02E‐02	1.51	LOC_Os05g25550	ATPase 7, plasma membrane‐type
Os.52968.1.S1_at	9.61E‐03	1.78	3.56E‐02	1.56	LOC_Os11g19160	Beta‐D‐xylosidase
Os.50337.1.S1_at	4.43E‐02	2.12	5.41E‐01	1.29	LOC_Os04g33720	Beta‐fructofuranosidase, insoluble isoenzyme 3 precursor
OsAffx.26489.1.S1_at	6.59E‐03	1.81	4.12E‐02	1.52	LOC_Os04g46760	Conserved hypothetical protein
OsAffx.28245.1.S1_at	2.32E‐02	1.54	2.66E‐02	1.52	LOC_Os07g03260	CSLC10—cellulose synthase‐like family C
Os.52482.1.S1_at	3.96E‐02	1.70	8.68E‐02	1.55	LOC_Os07g36630	CSLF8—cellulose synthase‐like family F; beta1,3;1,4 glucan synthase
Os.37822.2.S1_at	3.94E‐02	1.60	3.90E‐01	1.21	LOC_Os01g12030	Endoglucanase 1 precursor
Os.54770.1.S1_at	3.90E‐03	1.97	1.89E‐02	1.68	LOC_Os02g03120	Endoglucanase 1 precursor
Os.54812.1.S1_at	1.76E‐02	2.61	2.98E‐01	1.57	LOC_Os09g36060	Endoglucanase 1 precursor
OsAffx.3061.1.S1_x_at	4.89E‐02	1.71	1.31E‐01	1.50	LOC_Os02g54030	Endo‐polygalacturonase precursor
OsAffx.17383.1.S1_at	1.73E‐02	1.66	6.61E‐01	1.09	LOC_Os08g37750	Exo‐1,3‐beta‐glucanase
Os.5670.1.S1_at	4.15E‐02	1.66	7.43E‐02	1.55	LOC_Os08g23790	Exopolygalacturonase precursor
Os.8324.1.S1_a_at	3.32E‐02	1.61	3.40E‐02	1.61	LOC_Os01g66940	Fructokinase‐1
Os.12780.1.S1_at	3.31E‐02	1.60	1.57E‐01	1.35	LOC_Os08g02120	Fructokinase‐2
Os.24051.1.S1_at	5.27E‐03	1.78	3.47E‐01	1.19	LOC_Os04g33640	Glucan endo‐1,3‐beta‐glucosidase 7 precursor
Os.24051.1.S1_x_at	4.54E‐04	1.58	2.45E‐02	1.25	LOC_Os04g33640	Glucan endo‐1,3‐beta‐glucosidase 7 precursor
Os.24051.2.S1_at	4.89E‐04	1.58	8.32E‐02	1.18	LOC_Os04g33640	Glucan endo‐1,3‐beta‐glucosidase 7 precursor
Os.33745.1.S1_at	9.96E‐03	2.11	3.24E‐02	1.83	LOC_Os01g58730	Glucan endo‐1,3‐beta‐glucosidase GVI precursor
Os.53364.1.S1_at	3.53E‐02	2.26	7.75E‐01	1.14	LOC_Os07g40740	Heparanase‐like protein 3 precursor
Os.6114.1.S1_at	1.79E‐02	2.06	5.28E‐02	1.79	LOC_Os05g09500	Hexokinase‐1
OsAffx.4483.1.S1_at	1.25E‐02	1.66	6.50E‐02	1.42	LOC_Os05g31110	Hexokinase‐1
Os.3414.1.A1_at	2.74E‐02	1.65	2.07E‐01	1.32	LOC_Os08g40930	Isoamylase
OsAffx.29137.1.S1_at	2.41E‐02	1.53	1.73E‐01	1.28	LOC_Os08g10604	Pectinesterase‐1 precursor
Os.18244.1.S1_at	1.45E‐02	1.87	4.68E‐01	1.20	LOC_Os03g53790	Periplasmic beta‐glucosidase precursor
Os.9731.1.S1_at	3.06E‐02	1.68	1.71E‐01	1.38	LOC_Os12g36810	Polygalacturonase
Os.4879.1.S1_a_at	2.50E‐02	1.52	2.56E‐01	1.22	LOC_Os01g47550	Ribokinase
Os.26441.1.S1_s_at	3.29E‐02	1.70	2.69E‐02	1.74	LOC_Os11g45710	SFR2
Os.12725.1.S1_at	3.09E‐02	1.53	1.05E‐01	1.36	LOC_Os06g06560	Soluble starch synthase 1, chloroplast precursor
Os.49091.1.S1_at	2.52E‐02	2.05	6.12E‐02	1.82	LOC_Os05g05270	Sucrose phosphate synthase
Os.25677.1.S1_at	4.21E‐02	1.91	3.69E‐01	1.34	LOC_Os03g28330	Sucrose synthase 2
Os.57465.1.S1_x_at	4.79E‐02	1.52	2.95E‐01	1.24	LOC_Os03g28330	Sucrose synthase 2
Os.49763.1.S1_s_at	1.03E‐02	2.22	2.44E‐02	1.99	LOC_Os03g17230	UDP‐glucuronic acid decarboxylase 1
Membrane transport including sugar porter and ABCG						
Os.26786.1.S1_at	3.76E‐02	1.56	1.79E‐01	1.32	LOC_Os09g15330	Sugar transport protein 14
Os.50123.1.S1_x_at	1.05E‐02	1.89	4.97E‐02	1.59	LOC_Os07g10590	Sugar transport protein 8
Os.45939.1.S1_at	8.66E‐03	1.81	2.24E‐02	1.65	LOC_Os01g04190	Arabinose‐proton symporter
Os.45939.1.S1_x_at	2.72E‐02	1.55	7.65E‐02	1.40	LOC_Os01g04190	Arabinose‐proton symporter
Os.6624.1.S1_s_at	2.96E‐02	1.60	6.09E‐02	1.49	LOC_Os01g04190	arabinose‐proton symporter
Os.27138.1.S1_at	2.32E‐02	1.81	2.28E‐01	1.36	LOC_Os10g21590	Carbohydrate transporter/sugar porter
Os.54757.1.S1_at	3.41E‐02	1.71	1.11E‐01	1.49	LOC_Os03g05610	Inorganic phosphate transporter 1‐2
Os.50503.1.S1_at	2.47E‐02	1.70	7.45E‐02	1.51	LOC_Os03g04360	Inorganic phosphate transporter 1‐7
OsAffx.30403.1.S1_at	1.65E‐02	2.16	2.33E‐01	1.47	LOC_Os03g03680	Major facilitator superfamily protein
OsAffx.30403.1.S1_s_at	3.49E‐02	1.91	4.03E‐01	1.30	LOC_Os03g03680	Major facilitator superfamily protein
Os.53018.2.S1_x_at	3.65E‐03	1.73	1.46E‐02	1.53	LOC_Os04g43210	Proton myo‐inositol cotransporter
Os.31838.1.S1_at	2.10E‐02	1.79	6.71E‐02	1.57	LOC_Os03g24870	Solute carrier family 2, facilitated glucose transporter member 8
Os.26932.1.S1_at	1.38E‐02	1.88	3.65E‐01	1.25	LOC_Os07g37320	Sugar carrier protein C
Os.27613.1.A1_at	1.77E‐02	1.74	1.81E‐02	1.74	LOC_Os05g07870	Triose phosphate/phosphate translocator
Os.4704.1.S1_at	6.41E‐02	1.49	5.61E‐02	1.51	LOC_Os03g07480	Sucrose Transporter
Os.45486.1.S1_x_at	4.19E‐02	1.82	9.03E‐02	1.64	LOC_Os01g03144	ABC‐2 type transporter family protein
Os.46480.1.S1_at	4.86E‐02	1.65	1.97E‐01	1.38	LOC_Os10g35180	ATP‐binding cassette sub‐family G member 2
Os.11454.2.S1_at	3.22E‐02	1.58	9.25E‐02	1.42	LOC_Os01g34970	Multidrug resistance protein 8
Os.33212.2.S1_at	2.13E‐02	1.53	9.72E‐02	1.34	LOC_Os01g67580	Multidrug resistance‐associated protein 9
Os.51901.1.S1_at	7.71E‐03	1.91	2.19E‐02	1.71	LOC_Os02g21750	Multidrug resistance protein 4
Os.409.1.S1_at	5.28E‐03	1.58	2.32E‐02	1.41	LOC_Os07g15460	Metal transporter Nramp6
Os.25771.1.S1_at	1.70E‐02	1.68	1.88E‐02	1.67	LOC_Os01g65000	Ammonium transporter 2
Os.2678.1.S1_at	3.94E‐02	1.61	5.11E‐01	1.16	LOC_Os02g13870	Aquaporin NIP1.2
OsAffx.22646.1.S1_at	4.66E‐02	1.63	4.84E‐02	1.62	LOC_Os03g61290	ATCHX19
OsAffx.26700.2.S1_at	5.33E‐04	1.89	3.47E‐04	1.96	LOC_Os05g02870	ATPase, coupled to transmembrane movement of substances
OsAffx.26700.2.S1_x_at	3.42E‐04	1.86	2.07E‐04	1.94	LOC_Os05g02870	ATPase, coupled to transmembrane movement of substances
Os.46553.1.S1_at	4.92E‐02	1.73	7.97E‐02	1.62	LOC_Os10g13830	ATPase, coupled to transmembrane movement of substances
Os.46553.2.S1_x_at	3.69E‐02	1.76	4.96E‐02	1.70	LOC_Os10g13830	ATPase, coupled to transmembrane movement of substances
Os.24908.1.S1_at	1.06E‐02	1.83	3.31E‐02	1.63	LOC_Os08g39950	Potassium transporter 17
Os.25736.1.S1_at	4.22E‐02	1.58	2.57E‐01	1.28	LOC_Os09g31486	Heat shock 70 kDa protein, Mitochondrial precursor
Os.38164.1.S1_at	4.57E‐02	1.64	1.63E‐01	1.40	LOC_Os12g38180	heat shock cognate 70 kDa protein 2
Anther development						
Os.49681.1.S1_at	3.60E‐02	1.65	2.62E‐01	1.30	LOC_Os03g07140	Male sterility protein 2, DPW
Os.18429.1.S1_x_at	4.16E‐02	1.61	2.97E‐01	1.27	LOC_Os01g63580	Glycerol‐3‐phosphate acyltransferase 8
Os.49822.1.S1_at	1.51E‐02	1.75	2.75E‐02	1.64	LOC_Os06g11970	MADS‐box protein AGL66; MADS63
Os.53212.1.S1_at	1.20E‐02	1.70	4.93E‐02	1.48	LOC_Os04g21660	26S protease regulatory subunit 6A
Os.46480.1.S1_at	4.86E‐02	1.65	1.97E‐01	1.38	LOC_Os10g35180	ATP‐binding cassette subfamily G member 2
Os.20530.1.S1_at	2.01E‐02	1.59	2.19E‐01	1.26	LOC_Os08g44530	Dihydroxy‐acid dehydratase
Os.41468.1.S1_at	1.17E‐02	2.01	7.91E‐02	1.60	LOC_Os01g47050	Kelch motif family protein
Os.46553.1.S1_at	4.92E‐02	1.73	7.97E‐02	1.62	LOC_Os10g13830	ATPase, coupled to transmembrane movement of substances
Os.46553.2.S1_x_at	3.69E‐02	1.76	4.96E‐02	1.70	LOC_Os10g13830	ATPase, coupled to transmembrane movement of substances
OsAffx.12789.1.S1_s_at	9.09E‐03	2.35	8.85E‐02	1.72	LOC_Os03g08754	MADS‐box transcription factor 47
Os.50337.1.S1_at	4.43E‐02	2.12	5.41E‐01	1.29	LOC_Os04g33720	Beta‐fructofuranosidase, insoluble isoenzyme 3 precursor
Os.33948.1.S1_at	4.50E‐02	1.69	2.89E‐01	1.32	LOC_Os04g41110	N terminus of Rad21‐/Rec8‐like protein

**Figure 5:**
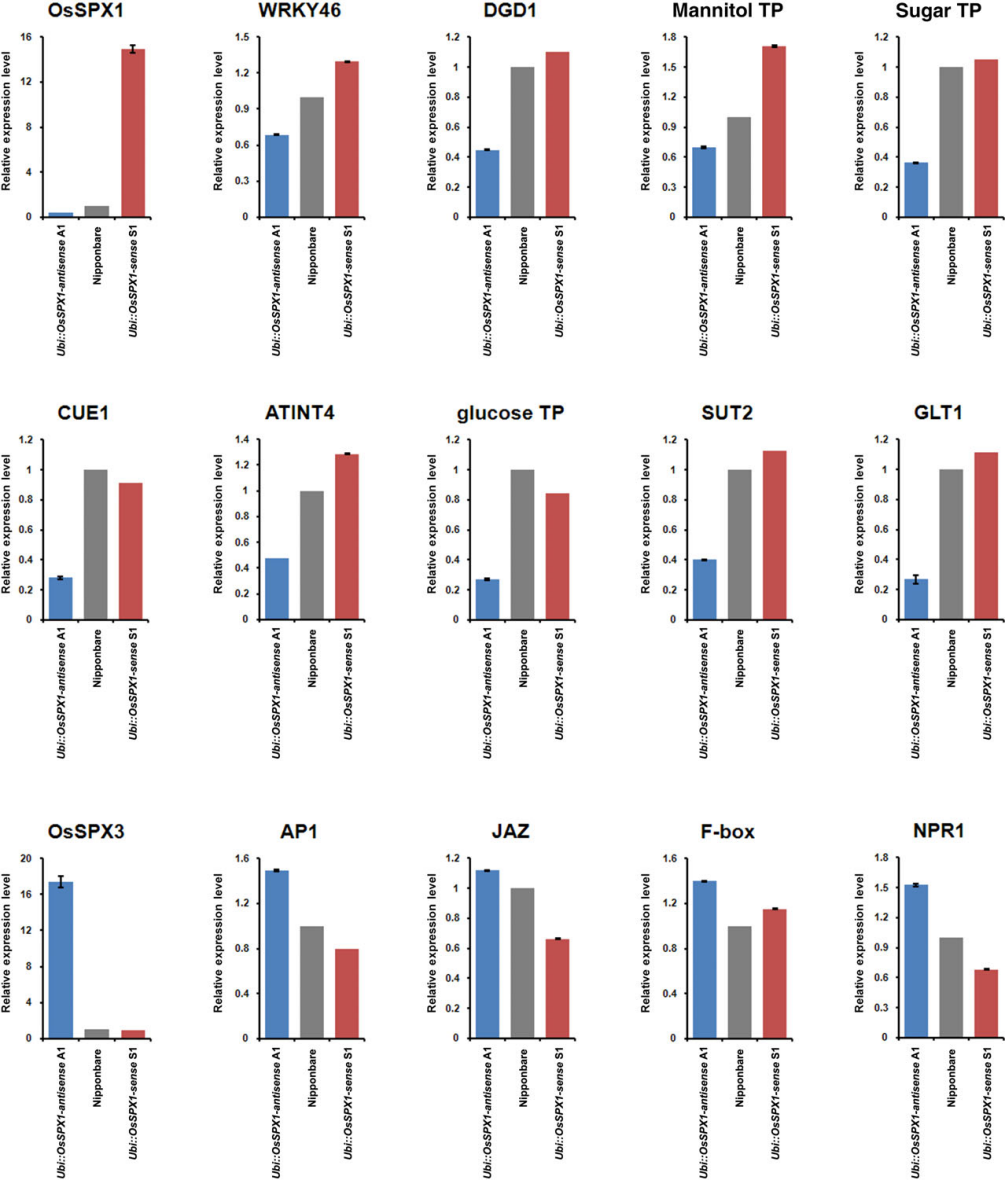
Real‐time RT‐PCR validation for selected probe sets in anthers. The probe sets were selected for real‐time RT‐PCR to validate the expression patterns among *Ubi::OsSPX1‐sense* transgenic line (red bar), WT (grey bar) and *Ubi::OsSPX1‐antisense* transgenic line (blue bar), the error bars represent the standard deviations of three replicates. The transcripts are as follows (**t**he primers for each probe set are listed in Table S2): OsSPX1—LOC_Os06g40120; IDS4‐like protein; SPX domain containing protein; WRKY46—LOC_Os12g02440; WRKY transcription factor 46; DGD1—LOC_Os04g34000; digalactosyldiacylglycerol synthase 1; mannitol TP—LOC_Os10g21590; carbohydrate transporter/sugar porter; sugar TP—LOC_Os07g10590; sugar transport protein 8; CUE1—LOC_Os05g07870; triose phosphate/phosphate translocator; ATINT4—LOC_Os04g43210; proton myo‐inositol cotransporter; glucose TP—LOC_Os03g24870; solute carrier family 2, facilitated glucose transporter member 8; SUT2—LOC_Os03g07480; sucrose transporter; GLT1—LOC_Os01g04190; arabinose‐proton symporter; OsSPX3—LOC_Os10g25310; SPX domain containing protein; AP1—LOC_Os07g01820; MADS‐box transcription factor 15; JAZ—LOC_Os07g42370; pnFL‐2; F‐box—LOC_Os10g41838; F‐box protein interaction domain containing protein; NPR1—LOC_Os01g09800; regulatory protein NPR1; BTBA1—Bric‐a‐Brac, Tramtrack, Broad Complex BTB domain with Ankyrin repeat region.

There were 15 genes selected for real‐time RT‐PCR validation (shown in Figure 5) based on the functional enrichment analysis of differentially expressed genes. Additional biological replicate anther samples were collected for real‐time RT‐PCR validation, and more than 90% of the tested genes confirmed the GeneChip results, especially for the seven sugar transporter genes (Figure 5). Compared to the GeneChip data, the fold change of genes analysed by real‐time RT‐PCR was not exactly same, but the change trends were similar.

## Discussion

Phosphorus is one of the major mineral nutrients for plant growth and development. Rice plants can accumulate abundant Pi in vegetative organs such as leaves at the early developmental stage and transport the Pi stored in the leaves to reproductive organs such as panicle at the late developmental stage. *OsSPX1* is involved in the Pi‐signalling network related to *OsPHR2* and *OsPHO2*. *OsPHR2‐Ov1* transgenic line showed disordered male reproductive organs. The *ltn1* (*OsPHO2*) mutant had significantly reduced tiller number and fertility compared to WT. We studied the potential function of *OsSPX1* during rice reproductive phase, and discovered that the *OsSPX1* antisense transgenic rice lines had lower seed‐setting rate. Thus, we further investigated the anther and pollen development of the transgenic and wild‐type plants and found that the pollen fertility was affected by antisense of *OsSPX1* gene, possibly through influence on pollen fertility. We conducted rice whole‐genome GeneChip analysis to compare the gene expression profiling between wild‐type and transgenic rice lines. GO and GeneBins analysis results showed that the down‐regulated genes in the *OsSPX1* antisense lines were significantly enriched in the following biological processes, including starch and sucrose metabolism, carbohydrate metabolism and sugar transport, anther development, cell cycle, etc.

During the pollen maturation, starch and lipids accumulate and the supply of photosynthetic assimilates (including sugar and lipids) from source organs is required (Goetz *et al*., 2001). In the functional enrichment analysis for the probe sets that had significantly lower expression in *Ubi::OsSPX1‐antisense* transgenic line A1, GeneBins, MapMan and GO analyses all highlighted the sucrose and starch metabolism and sugar transporter categories. Starch biosynthesis is critical during pollen maturation and sterile pollen are normally starch deficient (Datta *et al*., 2002). In maize, several key sugar metabolic genes were lower expressed in late pollen stage of CMS male‐sterile genotype than that of male‐fertile genotypes, including SPP (sucrose 6‐phosphate phosphohydrolase), IVR (invertase), HXK (Hexokinase), hexose transporter, etc. (Datta *et al*., 2002). In our results, two HXK genes (*LOC_Os05g09500* and *LOC_Os05g3111*) and one SPP homolog (*LOC_Os05g05270*) were significantly lower expressed in *Ubi::OsSPX1‐antisense* transgenic line A1 (Table 1). Moreover, a dozen of sugar transporter genes were also lower expressed in this line (Table 1). Expression patterns of seven sugar transporter genes were validated by real‐time RT‐PCR (Figure 5). For example, *LOC_Os10g21590*, a carbohydrate transporter/sugar porter gene has its *Arabidopsis* homologs, *AtPMT1* and *AtPMT2* more highly expressed in mature or germinating pollen grains, as well as in growing pollen tubes (Klepek *et al*., 2010). Analyses of reporter genes performed with promoter sequences showed expression in hydathodes and young xylem cells (both genes). For another gene of a sugar transport protein, *LOC_Os07g10590* – of, its *Arabidopsis* homologs, *AtSTP6* was only expressed during the late stages of pollen (Scholz‐Starke *et al*., 2003) and *AtSTP9* was specifically expressed in the male gametophyte (Schneidereit *et al*., 2003). In addition, several *ABCG* (ATP‐binding cassette transporter) genes were lower expressed in line A1 (Table 1), including *LOC_Os10g13830*, homolog of *Arabidopsis ABCG31*. It was reported that the many pollen grains in double mutant *abcg9/abcg31* were shrivelled up and collapsed when exposure to dry air (Schneidereit *et al*., 2003). These results indicate that antisense of *OsSPX1*, possibly through regulation of phosphate homoeostasis, affects the expression levels of some key genes related to carbohydrate metabolism and sugar transport, and then influences the transport of nutrients from source organs like flag leaves to sink organs like anthers.

Many genes were reported to be related to anther and pollen development both in rice and *Arabidopsis* (Wilson and Zhang, 2009). For example, both rice and *Arabidopsis* MIKC* type *MADS*‐box genes showed conserved expression in the gametophyte, while *OsMADS62*,* OsMADS63* and *OsMADS68* were all specifically expressed late in pollen development (Liu *et al*., 2013). In our result, the *OsMADS63* (*LOC_Os06g11970*) was significantly lower expressed in *Ubi::OsSPX1‐antisense* transgenic line A1 (Table 1). Another male sterility gene, *DPW* (*LOC_Os03g07140*), was also lower expressed in line A1. The mutant *dpw* was reported to show defective anther development and degenerated pollen grains (Shi *et al*., 2011).

Cell cycle process also plays an important role in the male gametophyte development (McCormick, 2004). The SPX protein in yeast, Pho81, is involved cyclin–cdk complex as a CDK inhibitor (Lee *et al*., 2000; Lenburg and O'Shea, 1996). There were several cyclin genes significantly lower expressed in *Ubi::OsSPX1‐antisense* transgenic line A1, including members of cyclins A, B and D, as well as some important regulator genes (Table S3). For example, in the transgenic RNAi rice lines of *LOC_Os02g40450*, reduced expression level of the *ROCK‐N‐ROLLERS* gene resulted in reduced fertility with partially sterile flowers and defective pollens (Chang *et al*., 2009). These data might indicate a connection between Pi starvation and cell cycle process, and their roles in male gametophyte development.

In addition, some phenylalanine metabolism pathway genes were affected by down‐regulation of *OsSPX1* and were highly expressed in line A1, including phenylalanine ammonia‐lyase (PAL), peroxidise, tropinone reductase, etc. (Table S3). Phenylalanine metabolism pathway is involved in the pollen development process. The transition of phenylpropanoids to flavonoids is considered as essential condition for viable pollen (Wiermann, 1970) and the *PAL* in tapetum cells of anthers might play an important role in pollen development (Kehrel and Wiermann, 1985). The activity of PAL protein was related to the number of fertile pollen grains at the flowering stage of broccoli (Kishitani *et al*., 1993).

In brief, we discovered a novel role of *OsSPX1* in rice pollen fertility and grain yield using the *OsSPX1* antisense and sense transgenic rice lines. Our results showed that antisense of *OsSPX1* caused rice semi‐male sterility and lower seed‐setting rate. We further conducted rice whole‐genome GeneChip analysis to elucidate the possible molecular mechanism and found that the enriched functional groups related to starch and sucrose metabolism, sugar porter, cell cycle, anther development, phenylalanine metabolism pathway, etc. Several genes related to male sterility and male gametophyte development were also lower expressed in *Ubi::OsSPX1‐antisense* transgenic lines, such as *DPW* and *ROCK‐N‐ROLLERS*. These results may help us to understand the possible novel functions of *OsSPX1* involved in rice reproductive development and grain yield.

## Experimental procedures

### Plant materials

Seeds of rice (Nipponbare as WT, and *Ubi::OsSPX1‐antisense* and *Ubi::OsSPX1‐sense* transgenic lines) were surface‐sterilized in 5% (w/v) sodium hypochlorite for 20 min and then washed in distilled water three or four times, then germinated in water for 2 day at room temperature and 1 day at 37 °C. The seedlings were planted in the paddy fields during the growing season in Beijing, China.

For phenotype evaluation: The spikelets of rice (Nipponbare as WT, and *Ubi::OsSPX1‐antisense* and *Ubi::OsSPX1‐sense* transgenic lines) were randomly collected at heading stage.

For RNA isolation: Anther samples were harvested from rice plants during heading stage under natural conditions in the paddy fields.

### Characterization of anther and pollen phenotypes

Anthers of the sampled flowers were dissected and immersed in Alexander's solution (Alexander, 1969). Stained pollen grains were released from anthers and observed under light microscopy (Zeiss, A1, Thuringia, Germany). For SEM, fresh anthers were coated with palladium‐gold in a sputter coater (Hummer), then observed and photographed by Hitachi S‐3400N scanning electron microscope (Hitachi, Japan). For TEM observation, the anthers were fixed in formaldehyde acetic acid using standard plastic sections and Hitachi JEM‐1230 (HC) transmission electron microscope (Hitachi, Japan) was used.

### RNA isolation and real‐time RT‐PCR

All anther and flag leaf samples from transgenic lines and WT were homogenized in liquid nitrogen before isolation of the RNA. Total RNA was isolated using TRIZOL^®^ reagent (Invitrogen, Carlsbad, CA) and purified using Qiagen RNeasy columns (Qiagen, Hilden, Germany). Reverse transcription was performed using Moloney murine leukaemia virus (M‐MLV; Invitrogen). We heated 10 μL samples containing 2 μg of total RNA, and 20 pmol of random hexamers (Invitrogen) at 70 °C for 2 min to denature the RNA and then chilled the samples on ice for 2 min. We added reaction buffer and M‐MLV to a total volume of 20 μL containing 500 μm dNTPs, 50 mm Tris–HCl (pH 8.3), 75 mm KCl, 3 mm MgCl_2_, 5 mm dithiothreitol, 200 units of M‐MLV and 20 pmol random hexamers. The samples were then heated at 42 °C for 1.5 h. The cDNA samples were diluted to 8 ng/μL for real‐time RT‐PCR analysis.

For real‐time RT‐PCR, triplicate quantitative assays were performed on 1 μL of each cDNA dilution using the SYBR Green Master Mix (PN 4309155; Applied Biosystems) with an ABI 7900 sequence detection system according to the manufacture's protocol (Applied Biosystems, Carlsbad, CA). The gene‐specific primers were designed using PRIMER3 (http://frodo.wi.mit.edu/primer3/input.htm). The amplification of 18S rRNA was used as an internal control to normalize all data (forward primer, 5′‐CGGCTACCACATCCAAGGAA‐3′; reverse primer, 5′‐ TGTCACTACCTCCCCGTGTCA‐3′). Gene‐specific primers were listed in Table S2. The relative quantification method (ΔΔCT) was used to evaluate quantitative variation between replicates examined.

### Affymetrix GeneChip analysis

For each sample, 8 μg of total RNA was used for making biotin‐labelled cRNA targets. All the processes about cDNA and cRNA synthesis, cRNA fragmentation, hybridization, washing and staining, and scanning followed the GeneChip Standard Protocol (Eukaryotic Target Preparation). In this experiment, Poly‐A RNA Control Kit and the One‐Cycle cDNA Synthesis kit were applied. Affymetrix GCOS software was used to do data normalization and comparative analysis.

In order to map the probe set ID to the locus ID in the rice genome, the consensus sequence of each probe set was compared by BLAST (Basic Local Alignment and Search Tool) against the TIGR Rice Genome version 5. The cut‐off e‐value was set as 1e–20. The singular enrichment analysis (SEA) tool in agriGO (Du *et al*., 2010) was applied for functional enrichment analysis of selected gene list, with the default parameters for Affymetrix Rice Genome Array. The functional enrichment analysis result was presented by REVIGO (Supek *et al*., 2011) tool with its default parameter for *Oryza sativa* GO term background. The gene function categorization was based on the functional classification BINs of *Oryza sativa* from MapMan (Thimm *et al*., 2004) and GeneBins (Goffard and Weiller, 2007).

## Supporting information


**Figure S1** Real‐time RT‐PCR validation of transgenic rice lines.

**Figure S2** Comparison of seed setting rate of *Ubi::OsSPX1‐antisense* transgenic lines, *Ubi::OsSPX1‐sense* transgenic lines and the WT rice in paddy fields.

**Table S1** 2403 probe sets showing differential expression in anthers among *Ubi::OsSPX1‐sense* transgenic line, WT, and *Ubi::OsSPX1‐antisense* transgenic lines.

**Table S2** Primer list of probe sets for real‐time RT‐PCR.

**Table S3** Selected differentially expressed probe sets related to cell cycle, chitinase, and phenylalanine metabolism.
