# Supplementary figures and images for: Down‐regulation of OsSPX1 caused semi‐male sterility, resulting in reduction of grain yield in rice
Source: Plant Biotechnol J. 2016 Jan 25;14(8):1661–72. doi: 10.1111/pbi.12527 (PMC5066639; doi:10.1111/pbi.12527)

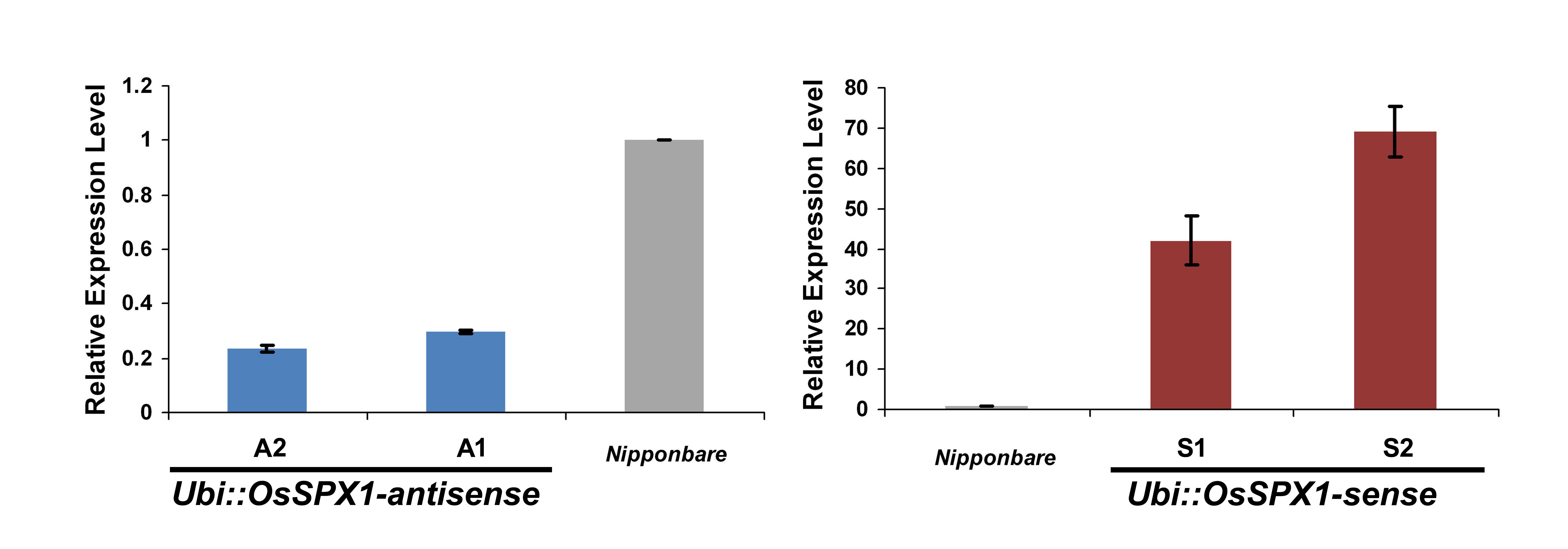

Supplement: Supplementary file 1 — Figure S1 Real‐time RT‐PCR validation of transgenic rice lines. [file PBI-14-1661-s004.jpg]

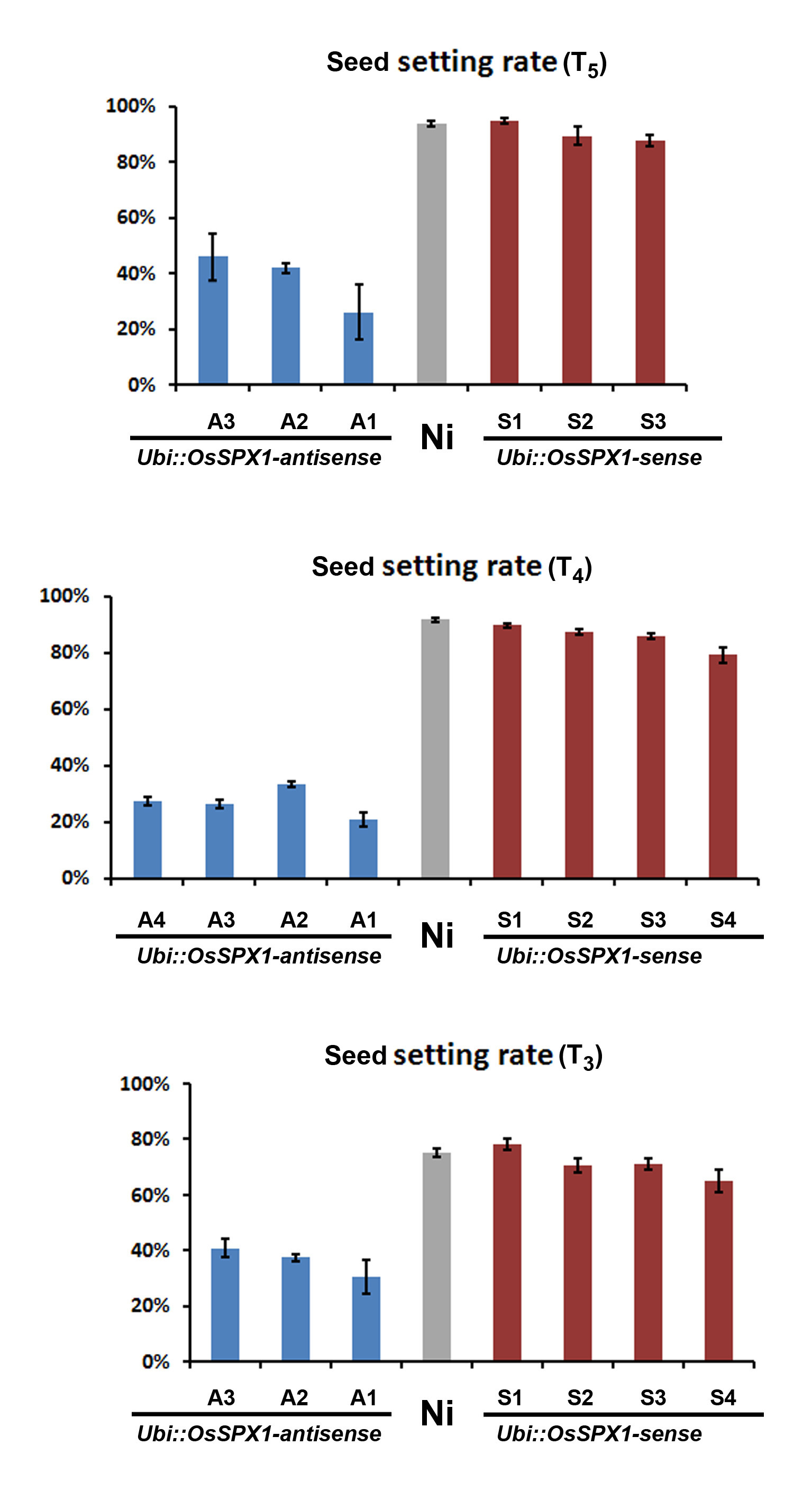

Supplement: Supplementary file 2 — Figure S2 Comparison of seed setting rate of Ubi::OsSPX1‐antisense transgenic lines, Ubi::OsSPX1‐sense transgenic lines and the WT rice in paddy fields. [file PBI-14-1661-s003.jpg]
