# Supplementary material for: Down‐regulation of OsSPX1 caused semi‐male sterility, resulting in reduction of grain yield in rice
Source: Plant Biotechnol J. 2016 Jan 25;14(8):1661–72. doi: 10.1111/pbi.12527 (PMC5066639; doi:10.1111/pbi.12527)
Supplement: Supplementary file 4 — Table S2 Primer list of probe sets for real‐time RT‐PCR. [file PBI-14-1661-s005.doc]

**Table S2. Primer list of genes for real-time RT-PCR**

| **Locus ID** | **Forward** | **Reverse** |
| --- | --- | --- |
| LOC_Os06g40120 (CDS region) ^(a)^ | GTCGCGGCGCTTAGGTCTC | TGCCGGTCGTGTCATTTG |
| LOC_Os06g40120 (UTR region) ^(a)^ | GTACACTGCATGACCTTGATCTTGA | CCGAGAGTTCATGAAAGAGGATGTAG |
| LOC_Os12g02440 | CACAGCCCATCACAGTATGG | GAACTTCATGGGCTTCGTGT |
| LOC_Os04g34000 | CAAGTCATTCCCCAACTGCT | GGGTCAAAgGTGAAGGTTCA |
| LOC_Os10g21590 | TTGAAATGGAAGGGACCTGA | CCACACGACACCAGAATGAC |
| LOC_Os07g10590 | ATCATGTGGGAACACGTGAA | TCGAGACGTAGACGCAGATG |
| LOC_Os05g07870 | CTTGCTGGCACTTGTTTTCA | AGTTGGCAACAGAATGGGTC |
| LOC_Os04g43210 | GGGGAGAAGGACTACAAGCC | TTTCATGGAACAGAGAGGCC |
| LOC_Os03g24870 | CCTTCGCGATTTCTTACTCG | ATCACCACGAACAGGATGGT |
| LOC_Os03g07480 | GTTGCCACCTCGGTAGAAGA | TACCAAGCACACTGCACACA |
| LOC_Os01g04190 | TGAGCGTCGTCAACAAATTC | TGGTCTCAACCACATTTCCA |
| LOC_Os10g25310 ^(b)^ | TCGCCGGACATGGAAAGGAT | CGGCGGCAGCGAGAACC |
| LOC_Os07g01820 | TGAGCCACCTCAATGCTTAA | AAGGTTGCTCCCATGATTCA |
| LOC_Os07g42370 | GAAGGCATCACTCCACAGGT | CATCTGAAGGAGAAGCCTGG |
| LOC_Os10g41838 | TGCTTAGCTTCTTCGGTCTTG | GCCTTGTCATTTGCAGGAGT |
| LOC_Os01g09800 | CAGTTTCTCTTGCAATGGCA | TAATCCTCGCCAAAGCAACT |

(a) The primer sequences from Wang et al., 2013; (b) The primer sequence from Zhao et al., 2009
