# Supplementary material for: Down‐regulation of OsSPX1 caused semi‐male sterility, resulting in reduction of grain yield in rice
Source: Plant Biotechnol J. 2016 Jan 25;14(8):1661–72. doi: 10.1111/pbi.12527 (PMC5066639; doi:10.1111/pbi.12527)
Supplement: Supplementary file 5 — Table S3 Selected differentially expressed probe sets related to cell cycle, chitinase, and phenylalanine metabolism. [file PBI-14-1661-s001.doc]

### Table S3. Selected differential expressed probe sets related to cell cycle, chitinase, and phenylalanine metabolism

| **Probe Set ID** | **S1 vs. A1** | | **Ni vs. A1** | | **Locus ID** | **Annotation** |
| --- | --- | --- | --- | --- | --- | --- |
|  | **p-value** | **Fold Change** | **p-value** | **Fold Change** |  |  |
|  |  |  |  |  |  |  |
| **Cell cycle and microtubule-based process** | | | | | | |
| Os.11022.1.S1_at | 2.62E-02 | 1.52 | 8.30E-02 | 1.37 | LOC_Os09g32526 | FK506-binding protein 2-1 precursor |
| Os.11022.1.S1_x_at | 8.03E-03 | 1.61 | 2.79E-02 | 1.45 | LOC_Os09g32526 | FK506-binding protein 2-1 precursor |
| OsAffx.30120.2.S1_s_at | 1.76E-02 | 1.59 | 7.45E-02 | 1.39 | LOC_Os09g32532 | 60S ribosomal protein L32 |
| Os.53132.1.S1_at | 7.90E-03 | 1.55 | 9.18E-02 | 1.28 | LOC_Os09g10300 | SKP1-like protein 1A |
| Os.11015.1.S1_at | 4.15E-02 | 2.29 | 1.17E-01 | 1.91 | LOC_Os03g56070 | expressed protein |
| Os.16227.1.S1_at | 1.84E-02 | 1.63 | 2.71E-01 | 1.24 | LOC_Os03g18380 | ubiquitin-activating enzyme E1 3 |
| Os.22281.1.S1_at | 3.09E-02 | 1.66 | 7.04E-02 | 1.52 | LOC_Os03g08754 | MADS-box transcription factor 47 |
| OsAffx.12789.1.S1_s_at | 9.09E-03 | 2.35 | 8.85E-02 | 1.72 | LOC_Os03g08754 | MADS-box transcription factor 47 |
| Os.24785.1.A1_at | 3.68E-02 | 1.50 | 1.33E-01 | 1.33 | LOC_Os01g70920 | cullin-1 |
| Os.27320.1.S1_at | 2.17E-02 | 1.60 | 1.32E-02 | 1.67 | LOC_Os11g05880 | protein binding protein |
| Os.31518.2.S1_x_at | 3.73E-02 | 1.71 | 2.25E-01 | 1.36 | LOC_Os01g17402 | cyclin-A2 |
| Os.31900.2.S1_x_at | 9.19E-03 | 1.51 | 4.48E-02 | 1.34 | LOC_Os07g23110 | poly synthetase 1 |
| Os.32564.1.S2_at | 1.63E-02 | 1.68 | 1.38E-01 | 1.35 | LOC_Os01g54420 | ATP binding protein |
| Os.32863.1.S1_at | 4.50E-02 | 1.57 | 3.05E-01 | 1.25 | LOC_Os05g33890 | microtubule-associated protein MAP65-1a |
| Os.33735.1.S1_at | 3.32E-02 | 1.65 | 6.12E-01 | 1.13 | LOC_Os08g02550 | COP9 signalosome complex subunit 3 |
| Os.33948.1.S1_at | 4.50E-02 | 1.69 | 2.89E-01 | 1.32 | LOC_Os04g41110 | N terminus of Rad21 / Rec8 like protein |
| Os.47376.1.A1_at | 3.87E-02 | 2.28 | 2.91E-01 | 1.56 | LOC_Os02g50630 | transcription regulator of the cell cycle TaE2Fe |
| Os.47981.1.S1_at | 4.79E-02 | 1.84 | 2.57E-01 | 1.43 | LOC_Os03g46920 | expressed protein |
| Os.48518.1.S1_at | 4.64E-02 | 1.54 | 4.50E-01 | 1.17 | LOC_Os01g67740 | mitotic chromosome and X-chromosome-associated protein mix-1 |
| Os.49557.1.S1_at | 5.13E-02 | 1.71 | 3.84E-02 | 1.77 | LOC_Os07g12630 | DNA binding protein |
| Os.49681.1.S1_at | 3.60E-02 | 1.65 | 2.62E-01 | 1.30 | LOC_Os03g07140 | male sterility protein 2, DPW |
| Os.49918.1.S1_at | 1.09E-02 | 1.62 | 7.57E-02 | 1.37 | LOC_Os02g02150 | ATP-dependent helicase yprA |
| Os.501.1.S1_at | 6.73E-03 | 1.52 | 1.91E-02 | 1.41 | LOC_Os01g15480 | EMB3013 |
| Os.50595.1.S1_at | 3.32E-02 | 2.21 | 2.09E-01 | 1.62 | LOC_Os03g39020 | kinesin heavy chain |
| Os.51585.1.S1_at | 6.35E-02 | 1.59 | 3.42E-03 | 2.21 | LOC_Os08g33488 | MADS-box transcription factor 23 |
| Os.51682.1.S1_at | 2.03E-02 | 1.70 | 1.05E-01 | 1.43 | LOC_Os03g02290 | ATP binding protein |
| Os.51936.1.S1_at | 4.03E-02 | 1.65 | 1.92E-01 | 1.37 | LOC_Os03g11380 | SNAP25 homologous protein SNAP29 |
| Os.52482.1.S1_at | 3.96E-02 | 1.70 | 8.68E-02 | 1.55 | LOC_Os07g36630 | CSLF8 - cellulose synthase-like family F; beta1,3;1,4 glucan synthase |
| Os.52764.1.S1_at | 4.79E-02 | 1.95 | 2.93E-01 | 1.44 | LOC_Os11g03200 | EMB2748 |
| Os.53823.1.S1_at | 6.13E-03 | 1.63 | 2.00E-02 | 1.48 | LOC_Os06g18830 | protein kinase G11A |
| Os.54253.1.S1_at | 4.48E-02 | 1.96 | 5.09E-01 | 1.27 | LOC_Os05g13300 | ATP binding protein |
| Os.54723.1.S1_at | 2.93E-03 | 1.81 | 3.83E-02 | 1.44 | LOC_Os03g25140 | peptidyl-prolyl isomerase PASTICCINO1 |
| Os.5643.1.S1_at | 7.27E-03 | 1.95 | 4.56E-02 | 1.60 | LOC_Os01g64900 | HEAT repeat family protein |
| Os.57434.1.S1_at | 4.71E-02 | 1.51 | 5.28E-01 | 1.14 | LOC_Os03g43684 | expressed protein |
| Os.9074.1.S1_s_at | 6.61E-03 | 1.68 | 1.74E-02 | 1.54 | LOC_Os03g02970 | endoribonuclease Dicer |
| Os.9246.1.S1_at | 3.18E-03 | 1.77 | 7.76E-03 | 1.64 | LOC_Os04g47580 | cyclin B2 |
| Os.9360.1.S1_at | 1.97E-02 | 1.66 | 9.51E-02 | 1.41 | LOC_Os12g40300 | skp1 family, dimerisation domain containing protein |
| OsAffx.18947.1.S1_x_at | 2.16E-02 | 1.51 | 7.29E-01 | 1.06 | LOC_Os11g19730 | expressed protein |
| OsAffx.19202.1.S1_at | 4.69E-02 | 2.32 | 5.63E-01 | 1.32 | LOC_Os11g36890 | conserved hypothetical protein |
| OsAffx.22073.1.S1_at | 3.37E-02 | 1.66 | 3.35E-01 | 1.25 | LOC_Os01g13310 | DNA topoisomerase 2 |
| OsAffx.22295.2.S1_at | 3.09E-03 | 2.34 | 1.01E-01 | 1.55 | LOC_Os07g33370 | IQ calmodulin-binding motif family protein |
| OsAffx.22295.2.S1_x_at | 9.61E-03 | 3.36 | 8.42E-02 | 2.30 | LOC_Os07g33370 | IQ calmodulin-binding motif family protein |
| OsAffx.23723.2.S1_at | 1.72E-03 | 2.27 | 3.82E-02 | 1.62 | LOC_Os01g49150 | syntaxin 72 |
| OsAffx.23866.1.S1_at | 3.41E-02 | 1.90 | 2.48E-01 | 1.42 | LOC_Os01g60050 | MOR1 |
| OsAffx.24187.1.S1_at | 1.50E-02 | 1.67 | 1.67E-01 | 1.31 | LOC_Os02g10490 | expressed protein |
| OsAffx.24661.1.S1_at | 4.15E-02 | 1.91 | 1.12E-01 | 1.65 | LOC_Os02g40450 | ROCK-N-ROLLERS |
| OsAffx.25614.1.S1_at | 2.80E-02 | 2.16 | 3.20E-01 | 1.44 | LOC_Os03g47820 | PINHEAD protein |
| OsAffx.29475.2.S1_s_at | 4.49E-02 | 1.91 | 4.40E-01 | 1.30 | LOC_Os08g32540 | cyclin delta-2 |
| OsAffx.29804.1.S1_at | 2.76E-02 | 1.98 | 3.39E-01 | 1.35 | LOC_Os09g11440 | expressed protein |
| OsAffx.29964.2.S1_at | 4.15E-02 | 1.80 | 9.52E-01 | -1.02 | LOC_Os09g21510 | mitogen-activated protein kinase kinase kinase 2 |
| OsAffx.9662.2.S1_at | 5.49E-03 | 2.52 | 2.15E-02 | 2.11 | LOC_Os09g14630 | retrotransposon protein |
| Os.53377.1.S1_at | 2.72E-02 | 2.24 | 2.21E-01 | 1.58 | LOC_Os08g28870 | receptor-like protein kinase 5 precursor |
| Os.48036.1.A1_at | 1.47E-02 | 1.79 | 5.59E-02 | 1.55 | LOC_Os09g15700 | receptor-like protein kinase 5 precursor |
| Os.47919.1.A1_s_at | 9.89E-03 | 1.66 | 1.26E-02 | 1.62 | LOC_Os05g33030 | kinesin-4 |
| Os.47919.1.A1_at | 4.14E-02 | 1.63 | 5.10E-02 | 1.59 | LOC_Os05g33030 | kinesin-4 |
| Os.27516.1.A1_at | 1.87E-02 | 1.63 | 2.67E-02 | 1.57 | LOC_Os09g24320 | 2-oxo acid dehydrogenases acyltransferase family protein |
| OsAffx.20042.1.S1_s_at | 4.71E-02 | 1.51 | 2.33E-01 | 1.27 | LOC_Os12g39980 | kinesin like protein |
|  |  |  |  |  |  |  |
| **Chitinase** | | | | | | |
| Os.1191.1.S1_at | 1.30E-03 | -2.21 | 1.93E-03 | -2.03 | LOC_Os03g04060 | basic endochitinase C precursor |
| Os.22000.1.S1_at | 3.32E-02 | -4.94 | 4.66E-02 | -3.63 | LOC_Os06g51060 | basic endochitinase 1 precursor |
| Os.22058.1.S1_at | 5.44E-03 | -1.52 | 2.64E-03 | -1.66 | LOC_Os10g39680 | acidic endochitinase Q precursor |
| Os.2692.1.S1_x_at | 3.79E-03 | -2.76 | 9.77E-03 | -2.08 | LOC_Os06g51050 | basic endochitinase 1 precursor |
| Os.27875.1.S1_at | 1.05E-02 | -1.79 | 2.36E-03 | -2.54 | LOC_Os10g28120 | chitinase 1 precursor |
| Os.28124.1.S1_at | 1.32E-02 | -8.32 | 1.33E-02 | -8.19 | LOC_Os10g28080 | chitinase 1 precursor |
| Os.32890.1.S1_at | 3.04E-02 | -1.97 | 1.07E-01 | -1.49 | LOC_Os11g47600 | xylanase inhibitor protein 1 precursor |
| Os.3415.1.S1_at | 3.21E-02 | -2.67 | 2.40E-02 | -3.09 | LOC_Os10g39680 | acidic endochitinase Q precursor |
| Os.37717.1.A1_s_at | 2.63E-02 | -2.25 | 2.62E-02 | -2.26 | LOC_Os05g15770 | xylanase inhibitor protein 2 precursor |
| Os.37717.1.A2_s_at | 1.41E-02 | -2.21 | 1.18E-02 | -2.33 | LOC_Os05g15770 | xylanase inhibitor protein 2 precursor |
| Os.51172.1.S1_x_at | 8.46E-03 | -2.67 | 1.65E-02 | -2.15 | LOC_Os06g51050 | basic endochitinase 1 precursor |
| Os.7991.1.S1_at | 4.64E-02 | -2.07 | 5.50E-02 | -1.96 | LOC_Os04g41620 | endochitinase A precursor |
|  |  |  |  |  |  |  |
| **Phenylalanine metabolism** | | | | | | |
| Os.10493.1.S1_at | 1.14E-02 | -3.35 | 5.54E-02 | -1.86 | LOC_Os02g43280 | aldehyde dehydrogenase 3B1 |
| Os.11547.1.S1_s_at | 7.60E-03 | -1.72 | 1.87E-02 | -1.51 | LOC_Os06g48030 | peroxidase 16 precursor |
| Os.11552.1.S2_at | 4.47E-02 | -1.98 | 3.88E-02 | -2.07 | LOC_Os04g59150 | peroxidase 12 precursor |
| Os.14965.1.S1_at | 1.36E-03 | -3.47 | 1.40E-03 | -3.42 | LOC_Os04g02754 | ATAMI1 |
| Os.15671.1.A2_at | 1.91E-02 | -1.54 | 6.76E-03 | -1.80 | LOC_Os12g39840 | expressed protein |
| Os.17232.1.S1_at | 9.81E-03 | -1.56 | 2.30E-01 | -1.15 | LOC_Os04g51310 | polyamine ABC transporter, periplasmic polyamine-binding protein |
| Os.17232.1.S2_at | 4.70E-02 | -1.91 | 1.34E-01 | -1.49 | LOC_Os04g51310 | polyamine ABC transporter, periplasmic polyamine-binding protein |
| Os.20079.1.S1_s_at | 1.63E-02 | -1.84 | 3.31E-02 | -1.62 | LOC_Os11g25160 | tropinone reductase 2 |
| Os.20079.1.S2_at | 1.23E-02 | -1.98 | 1.18E-02 | -2.00 | LOC_Os11g25160 | tropinone reductase 2 |
| Os.20118.1.A1_s_at | 1.27E-02 | -2.43 | 4.28E-02 | -1.75 | LOC_Os12g07150 | glutamyl-tRNA |
| Os.28397.1.S1_at | 2.07E-03 | -1.65 | 3.34E-04 | -2.26 | LOC_Os02g41650 | phenylalanine ammonia-lyase |
| Os.28397.1.S3_at | 1.24E-05 | -1.68 | 2.86E-06 | -2.08 | LOC_Os02g41650 | phenylalanine ammonia-lyase |
| Os.32471.1.S1_at | 4.62E-02 | -1.63 | 7.44E-02 | -1.50 | LOC_Os03g55410 | peroxidase 51 precursor |
| Os.38669.1.A1_s_at | 9.69E-03 | -1.50 | 3.99E-03 | -1.68 | LOC_Os11g42510 | tyrosine aminotransferase |
| Os.50838.1.A1_at | 3.48E-02 | -1.66 | 1.52E-01 | -1.32 | LOC_Os04g56180 | peroxidase 16 precursor |
| Os.5099.1.S1_at | 1.37E-02 | -1.59 | 1.23E-02 | -1.61 | LOC_Os10g39170 | peroxidase 1 precursor |
| Os.54937.1.S1_at | 2.69E-02 | -2.76 | 2.46E-02 | -2.88 | LOC_Os08g41780 | gastric triacylglycerol lipase precursor |
| Os.7935.2.S1_x_at | 4.09E-02 | -1.58 | 5.12E-02 | -1.53 | LOC_Os07g46852 | sex determination protein tasselseed-2 |
| Os.9698.1.S1_at | 5.04E-05 | -2.07 | 1.36E-04 | -1.77 | LOC_Os04g02780 | ATAMI1 |
| OsAffx.14625.1.S1_at | 3.07E-02 | -2.31 | 3.96E-02 | -2.12 | LOC_Os05g10780 | 1-aminocyclopropane-1-carboxylate synthase 7 |
| OsAffx.14625.1.S1_x_at | 1.08E-02 | -1.97 | 1.90E-02 | -1.76 | LOC_Os05g10780 | 1-aminocyclopropane-1-carboxylate synthase 7 |
| OsAffx.18586.1.S1_at | 4.12E-02 | -1.57 | 5.20E-02 | -1.51 | LOC_Os10g40020 | WW domain-containing oxidoreductase |
| OsAffx.28164.2.S1_at | 1.73E-02 | -1.53 | 3.54E-02 | -1.40 | LOC_Os06g48030 | peroxidase 16 precursor |
|  |  |  |  |  |  |  |
